# Biomarkers for Liquid Biopsies of Pituitary Neuroendocrine Tumors

**DOI:** 10.3390/biomedicines8060148

**Published:** 2020-06-02

**Authors:** Wilhelm Gossing, Marcus Frohme, Lars Radke

**Affiliations:** Division Molecular Biotechnology and Functional Genomics, Technical University of Applied Sciences Wildau, Hochschulring 1, 15745 Wildau, Germany; Wilhelm.gossing@th-wildau.de (W.G.); lradke@th-wildau.de (L.R.)

**Keywords:** pituitary, biomarker, liquid biopsies, cfRNA, ctDNA, miRNA, epigenetics, lncRNA

## Abstract

Pituitary neuroendocrine tumors (PitNET) do not only belong to the most common intracranial neoplasms but seem to be generally more common than has been thought. Minimally invasive liquid biopsies have the potential to improve their early screening efficiency as well as monitor prognosis by facilitating the diagnostic procedures. This review aims to assess the potential of using liquid biopsies of different kinds of biomarker species that have only been obtained from solid pituitary tissues so far. Numerous molecules have been associated with the development of a PitNET, suggesting that it often develops from the cumulative effects of many smaller genetic or epigenetic changes. These minor changes eventually pile up to switch critical molecules into tumor-promoting states, which may be the key regulatory nodes representing the most potent marker substances for a diagnostic test. Drugs targeting these nodes may be superior for the therapeutic outcome and therefore the identification of such pituitary-specific cellular key nodes will help to accelerate their application in medicine. The ongoing genetic degeneration in pituitary adenomas suggests that repeated tumor profiling via liquid biopsies will be necessary for personalized and effective treatment solutions.

## 1. Introduction

In recent years, reports of a high prevalence of adenomas in the pituitary gland have become more widespread in the literature, which gives rise to the impression that they may be more common than previously considered [1,2]. Several postmortem studies found a pituitary neuroendocrine tumor (PitNET) in 8–35% of randomly selected deceased people without recognized metabolic symptoms [3,4,5,6]. However, most studies cite Ezzat et al., who report an overall prevalence of 17% [7].

If PitNETs develop more frequently than assumed before, seemingly many of these never get detected or even noticed, since symptoms mostly emerge subtly over the course of many years. Eventually, this commonly leads to a late first diagnosis of 5–25 years after onset of symptoms, if at all, until which manifold health issues have manifested [2]. Earlier diagnoses could prevent many of these health problems. Therefore, it is important to improve the diagnostics of PitNET, which could furthermore initiate treatment for the many unnoticed cases.

Most adenomas remain benign, while—depending on the tumor secretory subtype—approximately 35% invade surrounding tissues [8]. Evaluating the invasiveness of a PitNET beforehand is important, as it coincides with tumor recurrence and resistance to medical therapies [9].

The causes that drive a pituitary adenoma to become invasive are not entirely clear yet. However, the authors Yang and Li break down the process of invasive transformation in PitNET into four key steps: (1) Induction of hypoxia-inducible factor 1α to tackle hypoxic conditions in growing tumor tissue. (2) Angiogenesis through overexpression of Vascular endothelial growth factor A (VEGFA). (3) Epithelial-mesenchymal transition (EMT) induced by overexpressed pituitary tumor transforming gene (PTTG1). (4) Degradation of the basement membrane and ECM by overexpressed matrix metalloproteinases (MMPs) [10]. Many of the markers investigated so far are linked to these four mechanisms and correlations with invasive PitNET have been shown in several studies [11,12,13,14,15,16]. However, misregulations of these molecules are not specific to the pituitary as they exist in other tumor types as well.

The ability to predict the progression of the tumor is still in need of improvement. Standard clinical practice methods include immunohistochemical staining for Ki-67 and p53 to assess the tumor aggressiveness upon diagnosis, but the definition of clinically relevant cutoffs is disputed and therefore not reliable enough for tumor prognosis [17].

Pituitary adenomas are routinely removed by transsphenoidal adenectomy. Using these solid biopsies for diagnostics only provides material from a single time point, which is insufficient for assessing its invasive capabilities since the tumors’ genotype is constantly evolving. Moreover, as only a small part of the tissue is used in the analysis, the whole genetic heterogeneity of the tumor is not captured, and crucial information may be missed. Nevertheless, most studies still focus on solid tissue research although the capabilities of liquid biopsies may offer some key advantages.

Liquid biopsies are easily accessible non-solid tissues such as blood, urine or saliva that enable repeated sampling at different stages of disease and may allow access to the complete genetic heterogeneity of tumors [18]. Such liquids are ready to be used on small and cheap point-of-care assays for a less elaborate analysis. Just like solid tissues, body fluids contain all individual clinical parameters that cover the entire range of epidemiological information, such as several types of RNA, DNA and proteins [19]. Liquid biopsies of different kinds have already been successfully used for the initial diagnosis as well as for prognostic information of pituitary tumors [20,21,22].

Due to their small size, pituitary tumors potentially release only relatively low titers of biomarkers into the bloodstream, so that detection systems have to be very sensitive. However, the pituitary gland is highly vascularized, which provides a good connection between its cells and the circulatory system [23]. Moreover, one demand is that the investigated biomolecules should pinpoint the original location of the tumor, paving the way for imaging and subsequent surgery. However, as most typical driver mutations occur in prominent oncogenes, they are often not tissue-specific. However, studies show that every tumor holds on average 80 different mutations and up to several hundred methylation changes [24]. Resulting epigenetic modifications may in turn change the expression of many types of non-coding RNAs or proteins directly, which can be just as momentous for the cell as a driver mutation. Finding such changes that reflect the properties and/or progression of a pituitary tumor is the most important aspect in search of a good biomarker. This review aims to assess the potential of looking for these different kinds of biomarker species in liquid biopsies since they have only been obtained from solid pituitary tissues so far.

## 2. Circulating Tumor DNA

Fragmented DNA released by dead tumor cells into the bloodstream is called circulating tumor DNA (ctDNA) and preserves all of its original genetic and epigenetic characteristics. A significant fraction of ctDNA is actively discharged within extracellular vesicles that protect their cargo from plasma nucleases [25]. Therefore, ctDNA could be a potent and easily available source for liquid biopsies of pituitary adenoma [26].

Due to its half-life of about 150 min, ctDNA always reflects an up-to-date image of the tumor [27]. Although the presence of cell-free DNA in the plasma is normal, tumors release elevated amounts that correlate with tumor weight [28]. Furthermore, malignant tumors release more ctDNA than benign ones [29]. However, the presence of a tumor cannot be inferred from cell-free DNA amounts only, as there exists a significant concentration overlap between healthy and diseased people [30].

Moreover, consecutive measurements of ctDNA concentration in plasma can give information about the progression of a tumor. While ctDNA levels decrease after tumor-resection, they were observed to increase upon cancer recurrence [31,32].

Specific driver mutations for PitNET-subtypes do exist and can be used to determine functionality or secretory subtype of the pituitary tumor. However, all the findings about mutations in PitNET, that have been gathered until now, originate from studies on solid pituitary tissues.

In Growth Hormone (GH)-producing PitNET, *GNAS* was found to be mutated in 30–53% of cases [33,34,35]. The mutation activates the cAMP-generating adenylyl cyclase, which increases GH production in these cells.

In adrenocorticotropic hormone (ACTH)-secreting pituitary adenomas, *USP8* mutations were found to be a main tumor-driving cause plus highly specific for this type of PitNET. These mutations accumulate in the 14–3-3 binding motif on exon 14 and occurred in 23–62% of ACTH-secreting PitNET [34,36,37]. Recently, two new recurrent mutations were found in the genes *BRAF* (17%) and *USP48* (23%) of corticotropic adenomas that could not be detected in other PitNET subtypes [38]. Both mutations enhance expression of the ACTH precursor Proopiomelanocortin (POMC), hence causing elevated ACTH secretion.

For prolactinomas, the overexpression of transcription regulation factor HMGA2 has been reported, which was mostly due to trisomy or amplification of the HMGA2 gene-bearing region on chromosome 12 [39,40,41]. HMGA2 upregulation was also observed in 67% of non-functioning pituitary adenomas (NFPA), but in these cases was not due to copy number variations (CNV) [42]. The expression of HMGA2 is normally absent in adult cells but can induce cell proliferation upon activation. Although its overexpression in PitNET is frequent, its well-known invasive potential in cancer is not observed.

In our own research, we sampled 25 patients with somatotropinomas and detected *GNAS* mutations in 54% of cases. However, *USP8* was also found mutated in 58% of cases, which is thought to be specific for ACTH-secreting adenomas (data not published).

Furthermore, two recurrent mutations could be linked to the invasiveness of PitNET. The PIK3CA protein is a prominent oncogene in many types of cancers with functions linked to cell survival and proliferation. *PIK3*CA-activating mutations have been detected in 8/91 (9%) malignant PitNET, but in 0/262 benign tumors, making this gene an indicator for invasive tumor progression [43].

The *MEN1* gene encodes a tumor-suppressing scaffold protein named menin. Mutations in *MEN1* entail a reduced binding of other functional proteins, leading to cell cycle dysregulations. Such mutations were found to be existent in ~3% of all PitNET. They are associated with a more aggressive behavior, a larger size and lower overall treatment response [44].

The total number of mutations occurring in a PitNET does not seem to be connected to its likeliness of invading surrounding tissue [45].

The high amount of genome-wide mutation studies among PitNET suggests that the few recurrent driver mutations we already know about are indeed the only ones. Finding novel subtype-specific hot-spot genes seems unlikely and the chance of them to be of broader diagnostic relevance is low.

Bi et al. report that they did not find any recurring mutations in 42 pituitary macroadenomas of different subtypes, not even a mutation in the *GNAS* or *USP8* gene [46]. When also considering that driver mutations may not be the sole cause of pituitary tumorigenesis, looking for mutations as diagnostic markers for PitNETs alone may not yield a sufficient sensitivity.

Tumors are often accompanied by copy number variations induced by chromosomal breaks in the area of tumor suppressor genes, by the doubling of tumor oncogenes or the formation of fusion genes. This can be caused by chromothripsis, a phenomenon that describes a clustered fragmentation and subsequent rearrangement of large parts of one or more chromosomes. Seemingly, it is not correlated with invasiveness or secretory subtype, but was found to be quite common among cancer entities [34,47,48].

In a screening of 125 PitNET, over 80% of samples showed CNVs of more than 10% of the genome, while 32% of samples even had more than 80% of their genome disrupted and loss of whole chromosomes was frequent [49]. Large disruptions of the genome were also found by Bi et al. in 42 PitNET, where 75% of functioning adenomas showed high disruptions and 87% of NF-PitNET showed low disruption [46]. This connection hints at CNV’s likely favoring oversecretion of pituitary tumors. The detection of chromothripsis may be of diagnostic importance, as it is linked to poor prognosis and therapy resistance in many types of cancer [50].

Although the presented studies show that DNA holds many information that could be beneficial for the diagnostics of PitNET, no studies have been conducted yet that tried to confirm these findings in ctDNA.

## 3. Cell-Free RNA

Another kind of nucleic acid that is available in liquid body compartments is cell-free RNA (cfRNA). cfRNAs often exert their actions through transcriptional, translational and functional regulation of DNA or proteins and except for mRNA only rarely code for proteins.

To date, the three subtypes messenger-RNA, long non-coding RNA and micro-RNA have shown correlations with PitNET, but there are only a handful of studies that have investigated them in liquid biopsies (Table 1). Just like ctDNA, cfRNA is actively packaged into extracellular vesicles of which exosomes carry the predominant part of cfRNA species that are interesting for liquid biopsies [51]. In addition, this makes them very stable in several body fluids, such as serum, plasma and urine [52]. Cell-free RNAs represent promising liquid biomarkers as their deregulation has been linked to tumorigenesis and tumor progression. They are of very special interest as a biomarker as their expression is strongly connected to specific cell types and thus can be used to combine tumor detection and localization.

### 3.1. Long Non-Coding RNAs

Most recently, long non-coding RNAs (lncRNAs) caught the researchers’ attention as they were found to exhibit the highest cell-type-specific expression differences [53]. They are non-protein coding transcripts of lengths greater than 200 nucleotides and can also be found in plasma, saliva or urine [54,55]. Extracellular lncRNA transport is mainly operated by exosomes, which adds RNase protection and stability [56].

A recent microarray analysis screened for >30,000 lncRNAs in 10 pituitary samples and found 113 differentially expressed lncRNAs that precisely clustered NF-PitNET and healthy samples. Further analyses identified the ten most different lncRNAs, of which only maternally expressed gene 3 (MEG3) was functionally described so far [57].

Several studies more confirmed the results for the lncRNA MEG3, whose expression levels are constantly decreased in PitNET and even more so in invasive adenoma. Interestingly, this reduction could only be observed in clinically non-functioning PitNET and was attributed to MEG3 gene promoter hypermethylation [58,59,60]. The expression of Hox transcript antisense intergenic RNA (HOTAIR) was found to be correlated to MEG3 levels and highly increased in invasive NF-PitNET, but also slightly increased in non-invasive PitNET [61]. However, the deregulations of MEG3 and HOTAIR are also prominent in other types of cancer and their value as a biomarker is therefore limited.

The lncRNA H19 is a well-known tumor marker and negatively correlated with tumor progression in PitNET [22]. It was found to be significantly reduced in PitNET samples and plasma exosomes. Furthermore, the expression of H19 did decrease with tumor volume [62]. Other research found higher levels of lncRNA H19 in invasive vs. non-invasive GH-secreting PitNET, which is inconsistent with the previous findings [63].

RPSAP52 is the complementary lncRNA to the high-mobility group AT-hook 2 (HMGA2) gene transcript, which is a known oncogene. RPSAP52 was recently found to be highly upregulated in 13 prolactinomas and gonadotropinomas. It enhances HMGA2 protein expression by sponging several miRNAs that target HMGA2 [64].

LncRNA colon cancer associated transcript 2 (CCAT2) is a promoter of cell proliferation and its upregulation is observed in several cancers. PitNET tissues were also found to overexpress CCAT2 in correlation to its invasive potential. It led to the overexpression of the oncogene PTTG1, thus contributing to tumor formation and progression [65].

Measuring the lncRNA IFNG-AS1 in 20 PitNET samples showed that it was upregulated and linked to invasiveness. By silencing IFNG-AS1 in vitro, the tumor progression could be inhibited, while its overexpression enhanced tumor growth. The effects were mediated through interaction with Epithelial splicing regulatory protein 2, which regulates the expression of FGFR2-IIIb [66]. IFNG-AS1 is also dysregulated in several human diseases and known to regulate Interferon-y, which has tumor-suppressive capabilities [67].

C5orf66-AS1 is a lncRNA that is expressed highest in the pituitary, but it is also associated with cancer in other cell types. It was found to be lower expressed in eleven NF-PitNET samples than four normal pituitary tissues and was moreover inversely correlated with invasive tumor behavior, indicating a tumor suppressive role [68].

In prolactinomas of 42 patients, the lncRNA clarin 1 antisense RNA 1 (CLRN1-AS1) was found to be downregulated. Normally, this lncRNA suppresses cell growth by sponging miR-217 which in its turn regulates the Wnt/β-catenin signaling pathway. It was further determined that the transcription factor Forkhead Box P1 is upregulated in these prolactinoma samples and suppressed expression of CLRN1-AS1 [69]. Besides PitNETs, CLRN1-AS1 is also associated with prognosis in hepatocellular carcinoma [70].

LncRNA SNHG1 is a potential marker for tumor progression as it was overexpressed in invasive pituitary tumor tissues. It acts as a sponge for the miRNAs miR-302, -372, -373 and -520, which all affect cell proliferation, migration and EMT [71]. It further inhibits tumor suppressors in other cancers as well [72].

Another study showed that the lncRNA X-inactive specific transcript (XIST) was upregulated in invasive PitNET samples against non-invasive and normal PitNET samples. This lncRNA silences miRNA-424-5p to increase the expression of basic fibroblast growth factor (bFGF), while elevated levels of miR-424-5p were shown to reduce proliferation and invasion in invasive PitNET cells [73]. Therefore, XIST can be used as predictor for invasive capabilities of PitNETs and other cancer types as well [74].

Plasmacytoma variant translocation 1 (PVT1) is a lncRNA that was found to be overexpressed in adenoma cells in vitro [75]. It promotes proliferation and EMT by activating several oncogenic signaling pathways and it does so by sponging more than 20 different miRNAs as well as being spliced itself into six different miRNAs that may affect tumorigenesis [76]. However, PVT1 upregulation was found in many cancer cell types, which argues for a more fundamental role in tumorigenesis and progression [77]. However, it also disallows any tissue specificity.

### 3.2. Micro-RNAs

Micro-RNAs (miRNAs) are short, non-coding RNAs, which intercept mRNA transcripts and thus can impede protein translation. It is estimated that more than 60% of all human genes are regulated by one or more miRNAs [78]. Thus, many known oncogenes are likely targeted by miRNAs, hence called oncomirs. They can be used in a liquid biopsy format, as they are circulating in the plasma after leaking from PitNET [79]. Moreover, they are resistant against RNases, temperature and pH changes, as they can be carried within exosomes or bound to proteins, rendering them well-suited for plasma screening tests [80,81]. In recent years, many studies have shown that miRNAs can directly promote pituitary tumorigenesis and predict their occurrence by looking for serum levels that differ from healthy individuals [82,83].

A study from Xiong et al. recently found elevated expression levels of the oncomiR miR-21-5p in exosomes and cells exclusively from GH-secreting PitNET. The exosomes with the miRNA were internalized by osteoblasts in vitro, leading to the induction of proliferation and differentiation by interfering with tumor suppressor *PDCD4*. These miR-21-5p containing exosomes from GH-secreting PitNET also increased bone formation in vivo and therefore may pose an alternative mechanism to the GH-induced growth increase that is linked to acromegaly [84]. It could furthermore explain the frequent ineffectiveness of somatostatin analogs used in certain acromegaly patients, which makes this type of miRNA an interesting diagnostic target.

Another miRNA was identified that is actively suppressing PTTG1 and thereby preventing tumorigenesis. This miRNA is called miR-423-5p and was found underexpressed in plasma samples of six somatotroph adenomas versus six healthy pituitary samples. In vitro experiments further showed that this miRNA is inhibiting cell proliferation and growth hormone release in GH3 pituitary cells, which makes it interesting for diagnostics and therapies [85].

The plasma levels of miR-143-3p were found to be significantly decreased exclusively in patients with gonadotropinomas after tumor removal. This resulted in an area under the ROC curve of 0.79, which grants this miRNA a diagnostic value for patient follow-up [86]. As this miRNA was also found to be reduced in early and late stages of colorectal tumors, it is assumed to be involved in tumor formation. Furthermore, a modified version of miR-143 showed an increased tumor-suppressive effect, which suggests therapeutic utility of this miRNA [87].

Further miRNA-based studies investigated only solid tissues, which is why their observed miRNA expression differences may be interesting to screen for in liquid tissues.

A feedback loop was uncovered that revolved around the oncogenes PTTG1 and p53 and the four miRNAs miR-300, miR-329, miR-381 and miR-655 [88]. These miRNAs also silence PTTG1 mRNA and were found to be downregulated in PitNET, leading to an overexpression of PTTG1. The expression of the four miRNAs is promoted by p53 and this activation was found to be blocked by PTTG1. A deregulation of these miRNAs can thereby induce a vicious cycle that overexpresses tumorigenic PTTG1.

Feng et al. gathered a large number of miRNAs that have been reported to be differentially expressed in either specific or multiple PitNET subtypes. Many of them target the transcription factors HMGA1 and HMGA2, which are known tumor suppressors diminishing pituitary cell proliferation. Their long list illustrates the heterogeneity of factors that can contribute to the formation of a tumor. While this complicates the choice of miRNAs to use for diagnostic purposes, miR-10b, miR-16, miR-26a, miR-503 and miR-514 have been found upregulated or downregulated in at least four different pituitary tumor subtypes and therefore could be superior markers for tumor screening [89]. On top of this, other studies found miR196a-2 and miR-212 to be deregulated in all pituitary tumor types. While miR196a-2 is also connected to HMGA2 regulation, miR-212 targets several genes involved in apoptosis [83,90]. For somatotropinomas, the miRNAs miR-125a-5p, miR-125b, miR-198, miR-503, miR-524-5p, miR-630 and miR-886-5p have been found upregulated or downregulated in 12 responders vs. three nonresponders to treatment with somatostatin analogs (SSA’s). Although the number of investigated patients is small, these miRNAs may represent valuable targets to evaluate on SSA therapy [91].

What is more, the differential expression of several miRNAs was correlated with the invasive potential of PitNET, thus giving prognostic information on tumor behavior. The levels of miR-15a, miR-16, miR-24, miR-25-3p, miR-34a, miR-93, miR-106b-5p, miR-132, miR-148-3p and miR-152 significantly differed in invasive PitNET in comparison to non-invasive ones [92,93,94,95,96,97]. Moreover, miR-137, which acts as a tumor suppressor by silencing the oncogene *AKT2*, was found significantly reduced in PitNET tissues. Its overexpression in vitro reduced the proliferation and invasiveness of pituitary tumor cells, while its suppression promoted these effects [98]. In several tumor types, hypermethylation of the promoter of miR-137 was found to be the cause for the reduced expression [99].

The miRNA-1299 was found to be connected to drug resistance in patients with prolactinomas by silencing FOXO1 expression. This micro-RNA showed reduced levels in drug responders versus nonresponders. The study further proved that FOXO1 inhibits prolactin expression by binding to its promoter, which makes FOXO1 mRNA and miR-1299 interesting diagnostic targets to predict drug response in patients with prolactinomas [100].

## 4. Epigenetics

Epigenetic alterations are key regulators of gene expression that may occur mainly through DNA methylation or histone modification. This influences the accessibility of affected sites for DNA- or RNA-polymerases and therefore also the genes’ transcriptional activity, which may lead to tumorigenesis. Usually, the number of epigenetic changes exceeds that of genetic mutations in a tumor by a factor of 10, which makes the landscape of epigenetic alterations more diverse, but individual markers less decisive [24]. Finding a single tumor-driving epigenetic marker with enough diagnostic sensitivity is therefore rather unlikely.

Methylation markers in liquid biopsies are tied to ctDNA, which retains its epigenetic characteristics after being released from its cellular origin [101]. The apparent advantage of analyzing methylation features is that they are more common among ctDNA fragments than somatic mutations. Although they can be used for detection or prognostic information of PitNET, they have yet to be investigated within liquid biopsies. Therefore, known pituitary methylation changes correlated with tumorigenesis and tumor invasiveness that may be interesting to screen for in liquid tissues will be illuminated in the following.

DNA methyltransferases (DNMTs) are the enzymes responsible for the maintenance of DNA methylations. Overexpression of two DNMT family members (DNMT1 and DNMT3A) was significant in macroadenomas and invasive PitNET and could be associated to hypermethylation of several tumor suppressor genes. In fact, DNMTs are frequently detected to be overexpressed in tumors and because they are connected to invasive tumor progression, DNMT inhibitors may be effective antitumor agents [102].

Hypermethylation in the promotor for *FGFR2* and corresponding low expression was found in 45% of human PitNET, which led to a reduction of tumor suppressor TP53 [103].

GADD45-γ expression was shown to be absent in 83% and 67% of PitNET, which was proven to be due to promoter methylation. The silencing could be reversed by treatment with the demethylating agent 5-Aza-2’-deoxycytidine in a human pituitary tumor cell line [104]. Interestingly, the induced re-expression of GADD45-γ in a pituitary tumor cell line attenuated tumor cell growth by 88% [105]. However, using GADD45-γ in liquid biopsies will not allow for localization of the tumor as it is already exploited in the treatment of other types of cancer [106].

Significant hypermethylation of the tumor suppressor retinoblastoma protein (RB1) promoter region in contrast to unmethylated normal postmortem pituitaries was detected in 60% of tested samples [107]. Further cell cycle regulating genes, such as *CDK1*, *CDKN1B*, *CDKN2A* and *CDKN2C* were found silenced and had hypermethylated promoters in PitNET tissue [108,109,110]. This common finding in sporadic PitNET is related to their biologic role, but they are probably not the inducers of tumorigenesis. Rather, they happen to appear often alongside tumor development and are therefore suboptimal candidates for early screening tests [111].

Neuronatin (*NNAT*) is a protein involved in brain development and formation of the nervous system. Its expression seems to be specific for the anterior pituitary gland, where its expression is the highest in the human body [112]. Transcript levels were found to be missing in 70% of PitNET independent of its subtype. As the underlying cause, CpG island methylation in the gene’s promoter region was determined. Furthermore, functional studies confirmed the inhibitory role of *NNAT* on cellular proliferation [113].

The universal tumor suppressor *RASSF1A* was also found to be silenced by promoter methylation in 50% of all subtypes of PitNET, except for gonadotropinomas with only 15% frequency. Promoter methylation was even more frequent in aggressive tumors, which hints at a role in tumor progression [114]. The role of *RASSF3* was investigated in 27 solely somatotroph adenomas versus four normal pituitaries and a significantly higher methylation level in its promoter as well as a corresponding lower mRNA expression was found in all samples [115]. Direct correlations of *RASSF3* in other cancer entities are not known, making this an interesting screening target.

In a large, genome wide study of the methylation pattern in pituitary adenoma, *HHIPL1* and *TFAP2E* were the two most significant candidates with 92% hypermethylation in PitNET than normal post-mortem pituitaries. Promoter methylation of *TFAP2E* was specific for NF-PitNET but is used as a predictor in the treatment of other types of cancer as well, whereas *HHIPL1*’s involvement in other malignancies is unknown [116].

In addition to this, epigenetic changes in methylation patterns can offer insights into malignant transformation of tumor tissues [117]. While malignancy is rare in PitNET, it is closely connected to invasiveness, which again is an important characteristic for pituitary tumor prognostics.

A large cluster of epigenetic hypomethylations connected to cell adhesion in six invasive PitNET could be shown in contrast to six non-invasive PitNET [118]. The overall methylation pattern in these invasive PitNET was significantly shifted towards hypomethylation. These findings support the key properties for malignant cell transformation that were proposed recently, as changes to the cell adhesion are important for the cell to be able to migrate and overcome the basal membrane [10]. Another study from 2014 could not find evidence for methylation differences in invasive and non-invasive NF-PitNET, however differences were skewed towards hypomethylation for functional (five somatotrophs) against 18 non-functional PitNET [119]. This finding is supported by research from 2018, where the authors could show that elevated gene expression levels of somatostatin receptor 5, growth hormone 1 and growth hormone 2 in GH-secreting PitNET also coincided with hypomethylations in the promoters of the respective genes [34].

Hypermethylation in the genes of cell adhesion proteins E-cadherin and T-cadherin are frequently seen in cancer. As such, promoter methylation was also found to be the reason for their reduced expression in 66% of PitNET and was correlated with their invasiveness [120].

The protein death associated protein kinase (*DAPK*) is a mediator of programmed cell death and loss of its expression was found in 59% of invasive PitNET samples, but only in 7% (1 of 15) of non-invasive samples. CpG island methylation in the promoter region of *DAPK* could be detected in 45% of these *DAPK*-negative samples, which makes it a good indicator of invasive progression [121].

## 5. Circulating Tumor Cells

Circulating tumor cells (CTCs) are cells spread from the original tumor tissue into the blood circulation. Their detection in patients with cancer is a matter of current research and may give treatment directions and information on prognosis. They are extremely rare in non-malignant diseases [122], which is the case for most PitNET. Furthermore, the isolation can be complicated due to their heterogeneous phenotype and a missing general marker for their identification. Considering their very low titer of about 1–5 CTCs per million normal blood cells together with the small size of a PitNET, CTCs do not have the best preconditions to be used for tumor detection [123].

Nevertheless, there is some ongoing research on using CTCs as diagnostic markers in the field of PitNET.

One study was able to detect CTCs in the interstitial vascular compartment of three patients with invasive PitNETs, which shows that also benign tumors can segregate CTCs [124]. Unfortunately, blood samples from these patients have not been tested for CTCs.

Another study only found CTCs in 60% of stage IV cancer samples, while they were absent in lower stages. Simultaneously, ctDNA could be found in all of the 16 samples from stages I—IV. In the blood samples that had CTCs, the ctDNA mutational count was over 50 times higher than in the CTCs alone [125]. This relation suggests, that the ctDNA is not derived from CTCs, but must have been shed from the primary source itself. What is more, the higher ctDNA load may better reflect the heterogeneous tumor DNA, thus providing more possible mutation sites to the analysis.

In conclusion, the benefit of using CTCs for the diagnostics of PitNET remains rather unclear while the presented comparison of ctDNA to CTCs suggests that molecular markers can be detected easier and in earlier cancer stages.

## 6. Exosomes

Most of the target substances circulating in blood are actively secreted by primary (tumor-) cells and transported via 40–100 nm sized vesicles called exosomes. Their molecular cargo consists of DNA, RNA and proteins that are embedded upon formation and conveyed throughout the body for intercellular communication. Exosomes have been successfully isolated from a multitude of body fluids, including blood plasma [126], urine [127] and saliva [128]. However, comparative studies between plasma-derived and urine-derived exosomes have seen a 100-fold lower number of exosomes in urine, while the total RNA was only 50% lower. Moreover, serum exosomes were found to contain much more miRNA in contrast to more rRNA in urine exosomes, which puts serum-derived exosomes in a better light [129]. pituitary-derived exosomes may contain molecular markers that can be extracted and analyzed for diagnostic and prognostic purposes as well as to monitor treatment response. Furthermore, cell surface receptors are encapsulated during their endocytotic formation that can be analyzed for localization purposes [19]. This means, that tumor markers detected within exosomes do not have to be tumor-specific but could still identify the type of tumor. This expands the useable pool of substances to more general and more frequent biomarkers like the TP53 mutation, which is why exosomes may be a very powerful tool for PitNET screening. This backtracking functionality was already shown to work with tissue-specific membrane proteins in patients with melanoma and patients with prostate cancer [130,131].

To date, there are mostly reports of different RNA types within exosomes that have been investigated for tumor diagnostics and prognostics [132]. In fact, several of the earlier described marker differences were exclusively derived and extracted from plasma exosomes.

As such, a study detected the exosomal mRNA expression profiles of several genes related to tumor progression and invasion in invasive and non-invasive NFPA. As a result, cyclin dependent kinase 6 (CDK6) and ras homolog family member U (RHOU) have been identified as potent biomarkers for predicting the tumor invasiveness in patients with NF-PitNET. The calculated sensitivity and specificity of the test were with 83% and 81% the highest when combining both markers [133].

However, reported expression differences have to be taken with care, as there is evidence that exosomal concentrations may not reflect the actual concentration in the tumor tissue because of selective loading. This conflictive expression has been shown in vitro for lncRNAs MALAT1, HOTAIR, lincRNA-p21, GAS5, TUG1 and CCND1-ncRNA [134].

## 7. Conclusions

Potential sources for pituitary tumor markers are manifold and comprise oncogenes, tumor suppressor genes, epigenetic alterations as well as expression changes of mRNA or proteins. A persistent hurdle for the identification of genetic and epigenetic biomarkers is the low abundance of ctDNA among circulating nucleic acids. The constant technological developments in, e.g., next-generation sequencing or digital PCR, however, have shown to be able to cope with this matter.

Recurrent mutations, such as in the genes *GNAS* or *USP8*, that are specific to PitNETs do exist and may well serve in co-diagnosing a secretory subtype. However, studies trying to detect them within body fluids have not been conducted yet and may be of good utility in future tumor screening tests.

While mutations occur randomly, methylations are controlled by enzymes and therefore happen at specific sites. Together with the greater number of aberrant methylations in PitNETs, the absolute chance of the cell to develop a tumor-inducing epigenetic change compared to a mutation is higher. This would furthermore explain the frequent absence of classic driver mutations in PitNETs and may hint at a superiority of epigenetic analyses over the screening for somatic mutations [135]. Moreover, methylation profiles are more recurring within the same tissue and therefore show better tissue-specificity [136].

The comparison of several biomarker species has been conducted by a study that performed ctDNA mutational analysis by targeted sequencing, methylome identification by targeted and whole-genome bisulfite sequencing, CNV analysis by whole-genome sequencing and cfRNA analysis by whole transcriptome sequencing in blood samples of patients with and without non-metastatic cancer. The authors concluded that the analysis of methylation patterns yielded the highest sensitivity for the detection of cancer with 65% in the non-metastatic group, while also having a specificity of 99%. For metastatic cancers, the detection rate rose to 95% [137]. Moreover, 90% of identified cancers could be correctly localized by means of the methylation data, allowing for a subsequent tumor removal [138]. Whether these results also apply for pituitary tumor detection has to be validated yet.

Interesting about epigenetic changes though, is their aspect of being reversible by using hypomethylating drugs, such as decitabine and azacitidine, to induce re-expression of hypermethylated genes. Thereby, detecting changes in methylation sites would simultaneously reveal possible targets for therapeutic options.

Furthermore, relationships of diagnostic relevance between miRNAs and PitNETs have been proven in many studies as well. There is a large variety of newly discovered differentially expressed miRNAs. However, such confirmed in both PitNET and liquid tissues remain scarce and so far only comprise miR-21-5p, miR-423-5p and miR-143-3p. It is conspicuous though, that none of the studies detected the differentially expressed miRNAs of the respective other studies. This also applies to the miRNAs from the studies with solid PitNET samples, meaning these miRNAs are seemingly not recurrent in patients with PitNETs. Hence, it can be deduced that the detected miRNAs are either “passenger alterations” and not involved in tumorigenesis or they are highly individual and therefore not suited as biomarkers for tumor screening.

Another class of cfRNAs that has been investigated in several more recent studies due to its assumed tissue-specific expression are the long non-coding RNAs. However, differential expression of presented lncRNAs is mostly not tissue-specific as detected differences are known to exist in other cancer types as well. H19 is a prominent oncogene in cancer and to this date the only lncRNA that has been investigated in body fluids in the context of PitNETs. While its correlation with tumor progression was verified in plasma exosomes, it may have a use in therapy assessment, but not in PitNET diagnostics. Concentration changes of other known cancer-associated lncRNAs could also be detected in PitNET tissues. However, the search for tissue-specific lncRNAs is important for liquid biopsies and lncRNAs are particularly promising as they often sponge several miRNAs at once. Thus, their dysregulation potentially affects several pathways simultaneously, which lifts their significance for tumorigenic processes.

With cfRNAs showing promising benefits as biomarkers, there are several more non-coding RNA species, such as circRNAs, snoRNAs and piRNAs, that have not yet been explored in the context of PitNETs. Concordant differential expression of these other species has already been confirmed in plasma and exosomes of cancer patients [139,140,141,142]. These findings illustrate the complex correlations between different RNA species and important cellular processes and that many of them may well play a role in the initial tumor formation.

**Figure 1:**
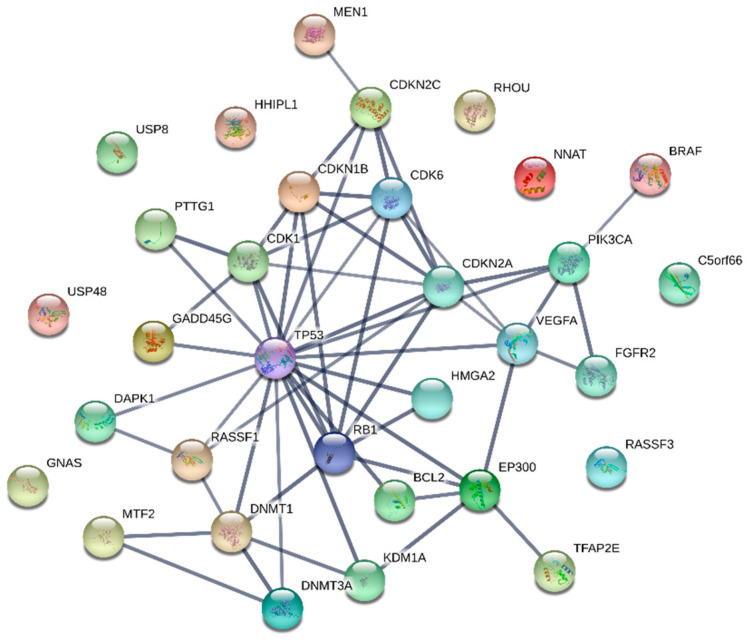
Interaction network of investigated marker molecules and their target proteins. Connected molecules may be less specific to the pituitary than separated ones.

Although the true nature of pituitary tumorigenesis has not yet been uncovered, the vast abundance of molecules already associated with the development of a PitNET suggests that it is probably due to cumulative effects of many genetic or epigenetic changes. They may slowly shift the expression of critical molecules that may be key regulatory nodes, to pathogenic levels. These critical molecules may represent the most potent marker substances for a diagnostic test, as minor changes pile up in them. Drugs targeting these nodes may consequently be superior for the therapeutic outcome. However, the overexpression of certain central molecules is prevalent in many types of cancers, which makes it important for liquid biopsies to screen for nodes specific to the pituitary. Many of the presented molecules interact with classical key proteins like TP53, RB1 or cyclin-dependent kinases and are known to play important roles in tumor development. Thus, they inherently lack specificity for the pituitary, which may limit their usefulness for liquid biopsies. Hence, identifying and investigating nodes outside of this interconnected network (shown in Figure 1) may yield less common tumorigenesis factors with greater pituitary specificity. Investigated molecules without connection to common key tumor proteins are *GNAS*, *USP8*, *USP48*, HHIPL1, *NNAT*, *RHOU*, C5orf66 and *RASSF3*, which seem to be more specific biomarkers and therefore should be validated for concordant differences in liquid biopsies.

While most studies using liquid biopsies investigate blood samples, urine is a rather unexplored body fluid for the diagnostics of PitNET. Cancer-specific mutations such as *K-Ras* and *PIK3CA* carried via circulating tumor DNA have been found in urine samples with higher sensitivity than in plasma or serum [143,144]. Using urine as a source for liquid biopsies has manifold advantages: it is completely non-invasive, it is a more homogeneous fluid with less downstream-processing inhibitors, it is easily obtainable at home without medical interference needed for sampling and larger volumes can be obtained which is advantageous rare event detection, for example. Therefore, the targeted search for markers of pituitary adenomas in urine samples may provide an attractive alternative to the current blood-based diagnostics.

With the advancements of more sensitive diagnostics and a growing awareness among physicians, it will become possible to improve the early diagnosis of pituitary tumors. Being able to choose the proper biomarkers is crucial to correctly assess early suspected cases. Simultaneously, the seemingly abundant unproblematic PitNETs that were revealed to exist by autopsy studies and that are not in need of treatment, may also become detectable. For these cases, it would be important to avoid gratuitous therapies, as they involve unnecessary risks and inconvenience for the patient.

In the end, the constant progression of genetic heterogeneity among pituitary adenomas suggests that in the future repeated tumor profiling via liquid biopsies will be unavoidable for effective tumor screening and treatment.

## Figures and Tables

**Table 1:** cfRNA biomarkers for pituitary neuroendocrine tumors (PitNET) that have already been detected in liquid biopsies.

Biomarker Type	Name	Tissue	Adenoma Subtype	Reference
lncRNA	H19	Plasma exosomes	all	10.1210/jc.2019-00536
miRNA	miR-143-3p	Plasma	gonadotropinomas	10.1210/jc.2018-02479
	miR-423-5p	Plasma	somatotropinomas	10.1155/2019/8516858
	miR-21-5p	Plasma exosomes	somatotropinomas	10.1016/j.trsl.2019.07.013
mRNA	Ras homolog family member U	Plasma exosomes	Non-functioning PitNET	10.24920/003585
